# Alterations in mechanical muscle characteristics and postural control induced by tennis match-play in young players

**DOI:** 10.7717/peerj.11445

**Published:** 2021-05-11

**Authors:** Joshua Colomar, Francisco Corbi, Ernest Baiget

**Affiliations:** 1 Institut Nacional d’Educació Física de Catalunya - INEFC Barcelona, Barcelona, Spain; 2 Academia Sánchez-Casal, Barcelona, Spain; 3 Institut Nacional d’Educació Física de Catalunya - INEFC Lleida, Lleida, Spain; 4 Sports Performance Analysis Research Group (SPARG), Universitat de Vic - Universitat Central de Catalunya, Vic, Spain

**Keywords:** Stiffness, Tone, Posture, Balance

## Abstract

**Background:**

Central and peripheric fatigue indicators are among the main reasons for performance decline following competition. Because of the impact of these factors on performance, how these variables are affected by match-play could be of interest, especially in young tennis players.

**Objective:**

This study aimed to investigate alterations induced by a simulated tennis match on postural control and muscle characteristics in young tennis players.

**Method:**

Seventeen male junior players took part in pre- and post-competition testing sessions performing postural control (displacement, speed and surface area of center of pressures) and muscle characteristics measurements (tone, stiffness, time to relaxation and elasticity). Between trials, participants played an 80-min simulated tennis match.

**Results:**

No significant differences were observed in any of the tested variables. Moderate-to-large effect sizes (ES) for decreased stiffness and tone and greater time to relaxation were found between pre- and post-conditions in the right rectus abdominis (−9.8%, −4.4% and 7.8%; ES = 0.8, 0.54 and −0.85). Also, a decrease in tone was found in the right vastus medialis (−7.1%; ES = 0.56), while surface area of the center of pressures with eyes open showed trends towards increasing in post-match measurements (55.9%; ES = 0.56).

**Conclusion:**

An 80-min simulated tennis match seems insufficient to elicit significant changes in postural control and mechanical muscle characteristics. Results suggest that physiological responses triggered by match-play were closer to those seen after a moderate activity than those present following a strenuous task.

## Introduction

An athlete’s capacity to recover and avoid overreaching is of great importance as it has clearly been related to risk of injury and overuse (Schwellnus et al., 2016). Competitive tennis requires players to participate in numerous matches, multiple draws and a high volume of events year-round. These matches are usually organized on consecutive days in professional players (Gescheit et al., 2015) and, although of shorter duration, can even take part on one same day when referring to younger players (i.e., U14, U16) (Gallo-Salazar et al., 2017). Although youth athletes seem able to recover faster than adults (Ratel et al., 2015), this particular organization model brings out certain needs when approaching recovery strategies specifically applied to the sport of tennis.

Many investigations have aimed to study the effects of match-play on physical and physiological responses. Research has typically focused on competition simulation following loads closer to highly-competitive tennis events (i.e., 2–4 h during consecutive days), concluding high values of perceived soreness, increased muscle damage, reductions in maximal voluntary contraction (MVC), decreased range of motion (ROM) and velocity production in main strokes (Gallo-Salazar et al., 2017; Gescheit et al., 2015; Martin et al., 2016). Although it seems clear that tennis match-play affects key performance factors, other fatigue indicators and monitoring tools could be important to approach recovery strategies effectively, especially when analyzing loads more often seen in junior tennis, with competition lasting around 1.5 h (Hoppe et al., 2014). Fatigue induced by exercise is determined by a combination of processes that affect not only peripheral but also central levels and result in decreased performance (Girard & Millet, 2009). Focusing on peripheral indicators, although muscle activity via electromyography (EMG) analysis has typically been studied to determine neuromuscular fatigue (Girard & Millet, 2009), muscle contractile characteristics quantified by tensiomyography measures (TMG) or shear wave elastography (SWE) have also been useful to determine the athlete’s fatigue status due to its lower cost and faster application ease and could be useful for overload detection and injury prevention (Sadeghi, Newman & Cortes, 2018). These techniques, as do newly developed equipment such as hand-held myometers, aim at analyzing the radial deformation of the muscle belly and the time it takes to happen during contraction caused by an external stimulation (De Paula Simola et al., 2016). This offers information about muscle displacement (Dm), tone, contractile force and response-time, all of which essential muscle mechanical properties involved in contraction. As stated in literature, an increased level of muscle stiffness and tone could interpose technical aspects due to reductions in ROM and increased peak forces (Colomar, Baiget & Corbi, 2020). In the same way, a non-sufficient level could inhibit the capacity to produce fast strokes (Roetert et al., 2009), due to the necessity of a leveled stiffness adjustment of the tendomuscular structure to effectively use elastic energy (Kuitunen et al., 2002). Thus, because of the impact of these factors on tennis player’s performance, how these variables are affected by tennis match-play could be of interest. Although some investigations have used TMG to detect exercise-induced fatigue (Wiewelhove et al., 2017), to our knowledge no studies have attempted to focus on stiffness or other muscle mechanical properties affected by competition in youth tennis players.

Moreover, other parameters regarding central activation capacities and neural drive indicators show a progressive activation deficit in prolonged tennis matches, increasing in the latter stages of exhausting competition (Girard & Millet, 2009). Alterations in postural control due to muscular fatigue have been reported in previous studies (Malliou et al., 2010) and seems a valid tool to assess fatigue at the central level (Paillard & Borel, 2013), as the nervous system is responsible of maintaining balance using information from the somatosensory, vestibular and visual inputs (Degache et al., 2014). When any of these channels modify the afferent information provided to the nervous system, balance and stability may be impaired (Ghram et al., 2019). For example, it’s well described how vision has a strong effect on postural control as this improves when increased visual information is provided (Ghram et al., 2016). In the same way, local muscle fatigue challenges the postural control system (Ledin, Fransson & Magnusson, 2004), evidencing an adaptive sensory reorganization (Vuillerme, Sporbert & Pinsault, 2009) in response to a given strenuous activity. To the best of our knowledge, few studies (Malliou et al., 2010) have attempted to focus on both, central and peripheric indicators of competition outcome, specifically in young tennis players, making interesting further knowledge around how load may affect performance, taking into account the particular organization models these athletes follow, which can typically lead to playing consecutive matches on the same day or throughout the week. Thus, the goal of this study was to investigate the alterations induced by tennis match-play on postural control and muscle mechanical characteristics.

## Materials & Methods

### Subjects

A priori power analysis for a difference between two dependents means was conducted in G-Power (version 3.1.9.5; University of Dusseldorf, Dusseldorf, Germany) to estimate a sufficient sample size. With the alpha level set at 0.05, using a target effect size (ES) of 0.8, a power of 0.80 and two tails, it was determined that a minimum sample size of *n* = 15 was required. Seventeen male junior tennis players (mean ± SD; age 16.5 ± 1.5 years; height 1.77 ± 0.07 m; body mass 69.3 ± 7.1 kg; BMI 22.2 ± 1.0 kg/m^2^) with an International Tennis Number (ITN) ranging from 2 to 4 (advanced level) and a weekly training volume of 25 h·week^−1^ volunteered for this study. Of these 25 h, 5 accounted for physical training, while 20 included technical and tactical sessions. One player performed a one-handed backhand while the remaining 16 players used a two-handed fashion. Also, one player was left-handed, while the remaining participants had a dominant right hand. Inclusion criteria for all subjects required each participant to have a minimum of 1-year experience in strength training and 5-years of tennis training and competition. Participants were excluded from the study if they had any upper body, back or knee pain as well as having surgery or participated in a rehabilitation process in the past 6 months. Before their participation, participants or their legal tutors in the case of being underage, voluntarily signed an informed consent. The study was conducted following the ethical principles for biomedical research with human beings, established in the Declaration of Helsinki of the AMM (2013) and approved by the Catalan Sports Council Institutional Review Board (19/2019/CEICEGC).

### Design

**Figure 1:**
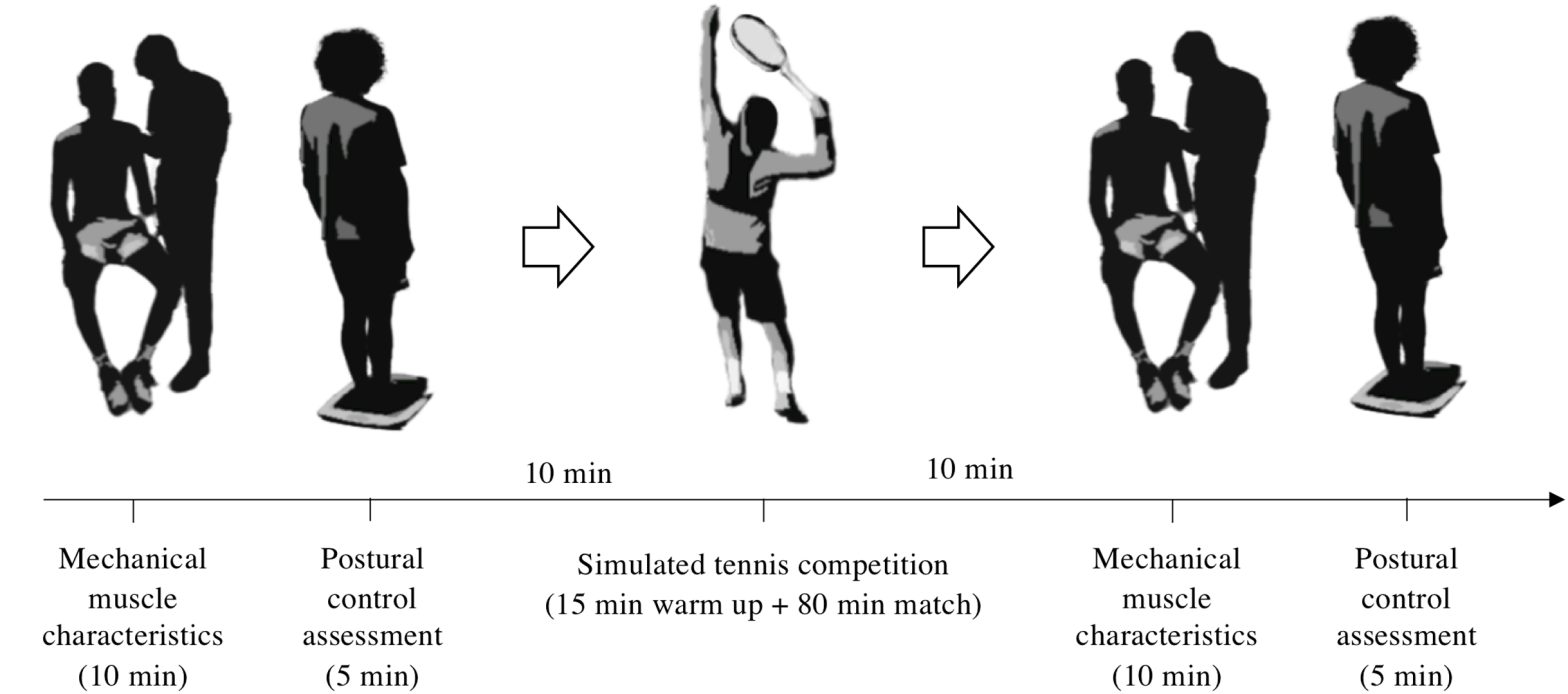
Schematic representation of the experimental procedure.

A cross-sectional repeated measures experimental design was carried by the same investigator in a static order in groups of two players. Each participant performed a pre-competition experimental trial and, 10 min later, took part in an 80-min simulated tennis match on an outdoor standard clay court following the International Tennis Federation (ITF) rules. After the match, participants immediately performed the post-competition testing session within a 10-min window (Fig. 1). Matches were undertaken by participants playing an opponent with a similar ranking or ITN and with one of the players wearing a GPS unit to register kinematic variables concerning match load. Before the match, players performed a 15-min standardized warm-up routine consisting of 5 min of joint mobilization, light jogging and rallying for an extra 10 min. The use of pain-relieving strategies (e.g., foam rolling, massage, ice baths, etc.) was not allowed in order to avoid interferences with the testing results. Players were allowed to consume water ad libitum. Isotonic and energetic drinks were not allowed during the simulated competition. Although only one of the players was wearing a GPS unit due to equipment availability, both participants took place in the pre-post experimental trials. This happened in eight of the simulated matches, while competition number nine was performed with both participants wearing the GPS device. Therefore, 10 participants were included in the match load analysis.

### Measurements

Participants were told not to exercise the day before the protocol took place, to maintain their habitual lifestyle during the study, to avoid excitatory substances (i.e., coffee or tea), and to consume their last meal at least 2 h before the scheduled test time. All measurements were obtained in the morning, approximately from 8 am to 10 am to avoid the influence of biorhythms on balance (Gribble, Tucker & White, 2007). All registrations were made within the competitive period.

#### Postural control assessment

Following Association Française de Posturologie standards (Bizzo, 1985), a posturographic platform (Fusyo-Medicapteur, Toulouse, France) at a 40 Hz sampling rate was used to assess postural control and data was recorded using Fusyo software (V1.2.1 - Medicapteur, Toulouse, France). The duration of each test was 51.2 s, resulting in a 2048-point time series. Participants were instructed as follows: ‘‘stand with your arms at your sides and look straight ahead while trying to maintain your stability the best you can’’ first with their eyes closed to, after, perform the test with their eyes open. They were instructed to stand double leg with arms at their sides and to look straight ahead. While having their eyes closed, subjects were asked to keep their gaze and to maintain postural stability. Only one postural test was performed in each condition (eyes open and eyes closed). To limit potential recovery time, no familiarization session was conducted.

#### Mechanical muscle characteristics

Muscle tone (mechanical tension in a relaxed muscle), stiffness (the resistance of the muscle to contraction or an external force that changes muscle’s initial shape), elasticity (recovery of the muscle’s initial shape after contraction or removal of an external force) and time to relaxation (time for the muscle to restore its initial shape after external force) (Ko et al., 2018) were recorded on both extremities using a hand-held myometer (Myoton-Pro®, Myoton AS, Tallinn, Estonia). The MyotonPro® measures the viscoelastic response of the muscle due to a brief (15 ms) mechanical impulse (force, 0.4 N) on the skin surface above the muscle (Julià-Sánchez et al., 2020). The mechanical deformation is delivered by the device testing end (d = 3 mm) held perpendicular to the skin surface (Treffel et al., 2016). The device was used in multi scan mode, where the mean value of five measurements was used for the reliability and statistical analysis. The assessment was conducted as in Colomar, Baiget & Corbi (2020). The muscles chosen were those mostly involved in tennis strokes (Correia et al., 2016; Kibler et al., 2007; Knudson & Blackwell, 2000), attending to the whole kinetic chain, measured in a relaxed state and in the following order: pectoralis major, biceps brachii, infraspinatus, deltoids, rectus abdominis, rectus femoris, vastus medialis, biceps femoris and the lateral head of the gastrocnemius.

#### Tennis match load

Matches were played for 80 min (Hoppe et al., 2014) regardless the score on a standard clay court under stable wind conditions (<2 m·s^−1^). Match load parameters of one of the players per match were recorded using a GPS unit (WimuPro®, Realtrack Systems, Almería, Spain). Variables included the number of high intensity accelerations (≥3 m·s^−1^) and decelerations (≥−3 m·s^−1^), accelerations and decelerations per minute, peak and mean running velocity and distance covered at different running intensities, as recommended in investigations with similar population (Gallo-Salazar et al., 2019; Hoppe et al., 2014). In order to register further data on match load, three heart rate zones were used for analysis. Using a heart rate monitor (Garmin HRM Dual Basic, Garmin, USA) and based on peak heart rate of the match (HR_max_), zone 1 was determined as low- (<70% HR_max)_, zone 2, moderate- (75–85% HR_max_) and zone 3, high-intensity- (>85 HR_max_), following literature (Gomes et al., 2011). After the match, general fatigue sensation for all participants was measured following the 6 to 20 Borg rate of perceived exertion (RPE) scale (Borg, 1982). Results concerning match load are summarized in Table 2.

### Statistical analysis

Descriptive data were reported as mean ± standard deviation (SD). The normality of the distributions and homogeneity of variances were assessed with the Shapiro–Wilk test. Intrasession reliability of measures was determined using a two-way average measure of the intraclass correlation coefficient (ICC), the standard error of measurements (SEM), and the coefficient of variation (CV) for each variable (Atkinson & Nevill, 1998). ICC, SEM and CV refer to intra-subject variation between 5 measurements. Parametric and non-parametric statistics were used when appropriate. Paired *t*-test were used to discern any significant differences between the mean values of pre- and post-measurements. Because some variables did not have a Gaussian distribution, Wilcoxon paired test was used. Mean differences in absolute and percent values were also used. The magnitude of the differences in mean was quantified as effect size (ES) and interpreted according to the criteria used by Cohen (1988) <0.2 = trivial, 0.2–0.4 = small, 0.5–0.7 = moderate, >0.7 = large. Seventy-eight preplanned comparisons were considered for this study. Accordingly, correction for multiple comparisons was undertaken using the Bonferroni method with a resulting operational alpha level of 0.0006 (*p* = 0.05/78). All statistical analyses were performed using SPSS 23.0 software (SPSS Inc., Chicago, IL, USA).

## Results

**Figure 2:**
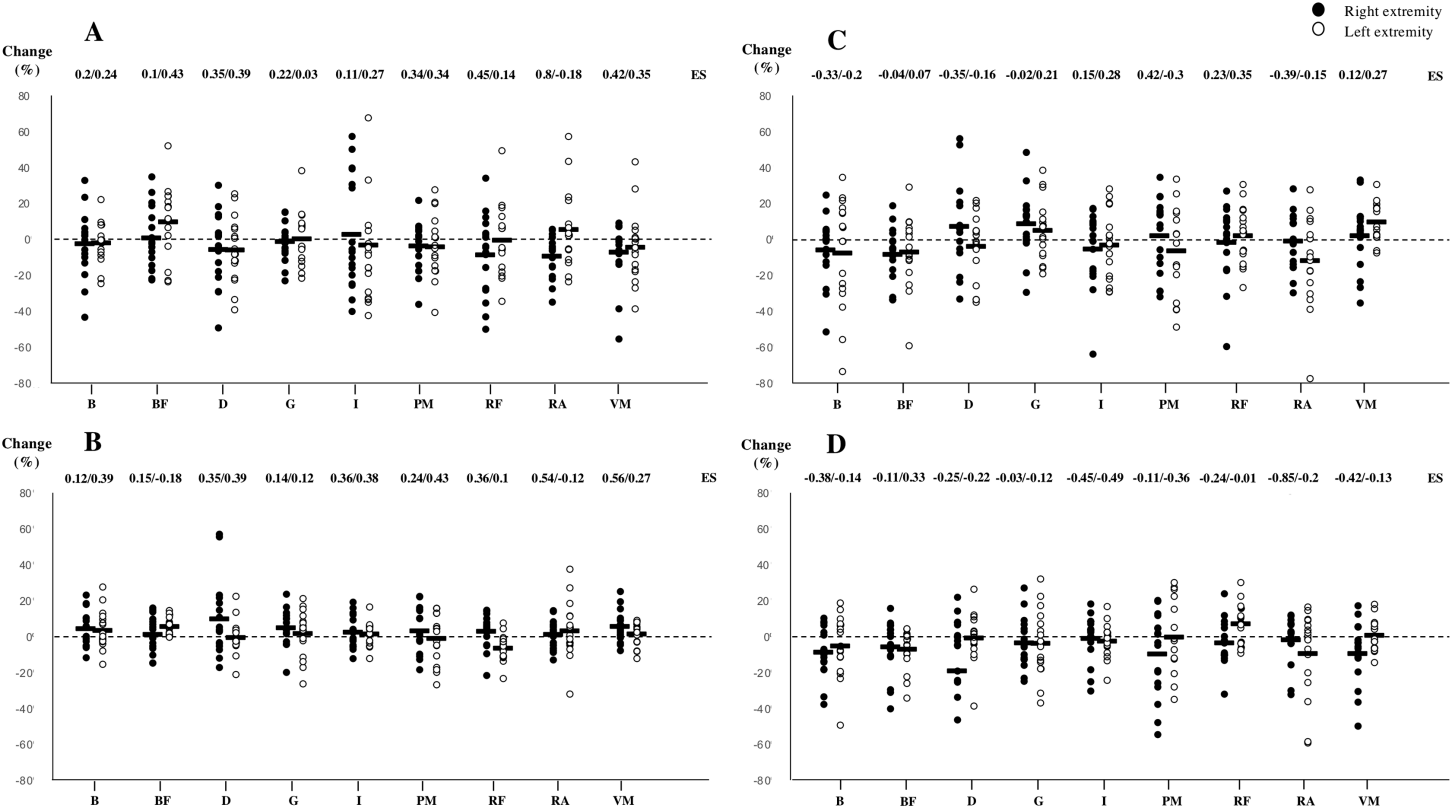
Changes produced in stiffness (A), tone (B), elasticity (C) and time to relaxation (D) from pre to post match measurements. ES, Cohen’s effect size; B, biceps brachii; PM, pectoralis major; D, deltoid; I, infraspinatus; RA, rectus abdominis; BF, biceps femoris; RF, rectus femoris; VM, vastus medialis; G, gastrocnemius. Bold line indicates mean value of the tested subjects (*n* = 17).

All of stiffness, tone, elasticity, time to relaxation and postural control measurements reached an acceptable level of reliability and are presented in Table 1. Mechanical muscle differences and postural control variations can be found in Tables 3 and 4. Effect sizes (ES) and percentage changes from pre to post match conditions are expressed in Fig. 2. No significant differences were observed in any of the tested variables. Non-significant moderate-to-large ES for decreased stiffness and tone and greater time to relaxation were found between pre- and post-conditions in the right rectus abdominis (−9.8%, −4.4% and 7.8%; ES = 0.8, 0.54 and −0.85) (Fig. 2). Also, a decrease in tone was found in the right vastus medialis (−7.1%; ES = 0.56) (Fig. 2 and Table 3). Total surface area with eyes open showed large ES towards increasing compared to pre-match measurements (55.9%; ES = 0.56) (Table 4).

**Table 1:** Reliability of test measurements (*n* = 17).

	Stiffness (N/m^−1^)				Tone (Hz)				Elasticity (Dm)				Relaxation (ms)		
	ICC [95% CI]	CV (%)	SEM		ICC [95% CI]	CV (%)	SEM		ICC [95% CI]	CV (%)	SEM		ICC [95% CI]	CV (%)	SEM
**Right BB**	0.998 [0.996–0.999]	1.5	3.4		0.998 [0.995–0.999]	0.9	0.17		0.997 [0.994–0.999]	2.1	0.03		0.995 [0.990–0.998]	1.2	0.25
**Left BB**	0.994 [0.987–0.997]	2.7	3.6		0.996 [0.993–0.999]	0.7	0.06		0.997 [0.993–0.999]	2.5	0.02		0.984 [0.967–0.994]	1.7	0.13
**Right BF**	0.998 [0.995–0.999]	1.9	7.1		0.998 [0.995–0.999]	1.2	0.13		0.988 [0.975–0.995]	3.5	0.03		0.998 [0.996–0.999]	1.8	0.23
**Left BF**	0.981 [0.961–0.992]	2.7	11.3		0.996 [0.991–0.998]	1.4	0.29		0.989 [0.978–0.996]	3.1	0.02		0.993 [0.985–0.997]	2.3	0.51
**Right D**	0.999 [0.998–1.000]	1.4	3.0		0.999 [0.998–1.000]	0.8	0.05		0.995 [0.989–0.998]	2.7	0.02		0.999 [0.998–1.000]	1.2	0.13
**Left D**	0.994 [0.988–0.998]	2.5	3.2		0.997 [0.994–0.999]	0.8	0.07		0.983 [0.965–0.993]	3.4	0.05		0.998 [0.996–0.999]	1.5	0.27
**Right G**	0.999 [0.999–1.000]	1.6	2.8		0.999 [0.998–1.000]	0.9	0.11		0.995 [0.991–0.998]	2.2	0.01		1.000 [0.999–1.000]	1.4	0.19
**Left G**	1.000 [0.999–1.000]	1.2	2.0		1.000 [1.000–1.000]	0.6	0.10		0.998 [0.996–0.999]	2.6	0.02		0.999 [0.998–1.000]	1.0	0.13
**Right I**	1.000 [0.999–1.000]	2.0	4.5		0.999 [0.999–1.000]	0.8	0.10		0.998 [0.996–0.999]	2.8	0.02		0.998 [0.995–0.999]	1.8	0.15
**Left I**	0.999 [0.999–1.000]	2.5	2.9		0.999 [0.999–1.000]	0.8	0.05		0.996 [0.992–0.998]	3.2	0.01		0.998 [0.996–0.999]	1.4	0.19
**Right PM**	0.998 [0.996–0.999]	2.5	4.4		0.999 [0.998–1.000]	1.0	0.21		0.997 [0.994–0.999]	2.5	0.02		0.996 [0.991–0.998]	2.1	0.30
**Left PM**	0.997 [0.994–0.999]	2.2	2.8		0.998 [0.996–0.999]	0.8	0.15		0.996 [0.992–0.998]	2-5	0.02		0.995 [0.990–0.998]	1.7	0.47
**Right RF**	0.999 [0.998–1.000]	1.5	5.8		0.999 [0.998–1.000]	0.8	0.13		0.996 [0.992–0.999]	3.5	0.02		0.999 [0.998–1.000]	1.1	0.18
**Left RF**	0.999 [0.999–1.000]	1.4	4.6		0.999 [0.999–1.000]	0.8	0.18		0.996 [0.992–0.998]	3.9	0.03		0.999 [0.999–1.000]	1.1	0.14
**Right RA**	0.997 [0.994–0.999]	2.9	5.5		0.997 [0.995–0.999]	1.7	0.24		0.993 [0.986–0.997]	3.8	0.04		0.996 [0.992–0.998]	2.6	0.22
**Left RA**	0.998 [0.997–0.999]	2.7	6.5		0.998 [0.996–0.999]	1.8	0.24		0.995 [0.990–0.998]	3.5	0.03		0.998 [0.997–0.999]	2.1	0.40
**Right VM**	0.999 [0.998–1.000]	1.6	1.8		0.999 [0.998–1.000]	0.8	0.03		0.997 [0.994–0.999]	3.5	0.03		0.999 [0.998–1.000]	1.3	0.24
**Left VM**	0.998 [0.997–0.999]	1.9	5.3		0.999 [0.999–1.000]	0.6	0.09		0.996 [0.991–0.998]	3.2	0.01		0.998 [0.995–0.999]	1.2	0.25

**Note:**

CV, coefficient of variation; ICC, intraclass correlation coefficient; SEM, standard error of measurement; Dm, decrement; BB, biceps brachii; PM, pectoralis major; D, deltoid; I, infraspinatus; RA, rectus abdominis; BF, biceps femoris; RF, rectus femoris; VM, vastus medialis; G, gastrocnemius.

**Table 2:** Match load characteristics (*n* = 10).

Variables	Mean ± SD
Total distance (m)	3961.8 ± 437.4
Distance at 0 to >1 m·s^−1^ (m)	1309.4 ± 84.2
Distance at 1 to >2 m·s^−1^ (m)	2014.5 ± 404.0
Distance at 2 to >3 m·s^−1^ (m)	407.5 ± 85.9
Distance at 3 to >4 m·s^−1^ (m)	155.3 ± 42.7
Distance at ≥4 m·s^−1^ (m)	75.2 ± 23.9
Peak velocity (m·s^−1^)	5.95 ± 0.44
Mean velocity (m·s^−1^)	1.05 ± 0.07
High intensity accelerations (≥3 m·s^−1^; n)	39.7 ± 10.1
High intensity decelerations (≥ −3 m·s^−1^; n)	57.9 ± 14.0
High intensity accelerations (≥3 m·s^−1^; n·min^−1^)	0.51 ± 0.12
High intensity decelerations (≥−3 m·s^−1^; n·min^−1^)	0.76 ± 0.18
Peak HR (beats·min^−1^)	184.0 ± 8.4
Mean HR (beats·min^−1^)	144.7 ± 12.5
% time spent at 0–70% of maximum session HR	20.9 ± 15.2
% time spent at 70–85% of maximum session HR	43.5 ± 10.3
% time spent at 85–100% of maximum session HR	32.5 ± 18.0
RPE	15.2 ± 1.1

**Note:**

HR, heart rate; n·min^−1^, number of actions per minute; RPE, rate of perceived exertion.

**Table 3:** Pre and post-match mechanical muscle characteristics scores (*n*=17).

	Stiffness		Tone		Elasticity		Relaxation	
	**PRE** **(N/m**^**−1**^**)**	**POST** **(N/m**^**−1**^**)**	**PRE** **(Hz)**	**POST** **(Hz)**	**PRE** **(Dm)**	**POST** **(Dm)**	**PRE** **(ms)**	**POST** **(ms)**
**Right BB**	203.3 ± 29.9	195.4 ± 33.6	13.8 ± 1.1	13.6 ± 1.3	1.1 ± 0.2	1.2 ± 0.2	21.7 ± 1.9	22.5 ± 2.3
**Left BB**	187.5 ± 23.0	181.9 ± 18.6	12.9 ± 0.8	12.6 ± 0.9	1.1 ± 0.2	1.1 ± 0.2	23.4 ± 2.4	23.7 ± 2.0
**Right BF**	410.0 ± 93.7	402.9 ± 66.9	19.9 ± 2.7	19.6 ± 2.3	0.9 ± 0.2	0.9 ± 0.2	12.7 ± 2.7	12.9 ± 23
**Left BF**	361.5 ± 63	392.1 ± 78.3	18.7 ± 2.0	19.0 ± 2.1	0.9 ± 0.2	0.9 ± 0.2	14.0 ± 2.2	13.3 ± 26
**Right D**	252.8 ± 45.8	232.2 ± 39.0	15.4 ± 1.4	14.7 ± 1.3	0.9 ± 0.1	0.9 ± 0.1	19.3 ± 2.7	20.1 ± 27
**Left D**	234.2 ± 39.1	216.2 ± 42.9	15.1 ± 1.3	14.5 ± 1.2	0.8 ± 0.1	0.8 ± 0.1	18.6 ± 2.7	19.2 ± 2.3
**Right G**	322.4 ± 52.8	314.0 ± 34.9	18.3 ± 2.0	18.0 ± 1.5	0.9 ± 0.1	0.9 ± 0.1	16.4 ± 2.7	16.5 ± 1.9
**Left G**	316.6 ± 45.3	314.9 ± 52.5	18.1 ± 2.0	17.9 ± 2.0	0.9 ± 0.1	0.9 ± 0.1	16.7 ± 2.7	17.0 ± 3.0
**Right I**	209.0 ± 92.8	201.9 ± 69.0	15.4 ± 4.6	13.8 ± 1.9	0.7 ± 0.2	0.7 ± 0.1	19.3 ± 4.3	20.9 ± 3.0
**Left I**	230.8 ± 61.4	211.5 ± 53.6	16.0 ± 5.2	14.2 ± 1.5	0.7 ± 0.2	0.7 ± 0.1	18.9 ± 3.9	20.8 ± 3.0
**Right PM**	175.0 ± 28.0	165.7 ± 21.9	12.1 ± 1.1	11.9 ± 0.8	1.1 ± 0.2	1.0 ± 0.1	25.1 ± 2.8	25.3 ± 2.5
**Left PM**	197.0 ± 33.5	184.7 ± 24.2	13.1 ± 0.9	12.6 ± 1.1	1.0 ± 0.2	1.0 ± 0.2	23.2 ± 2.8	24.2 ± 2.9
**Right RF**	338.9 ± 70.4	302.3 ± 79.5	17.5 ± 2.2	16.6 ± 2.2	0.9 ± 0.2	0.9 ± 0.2	15.3 ± 3.1	16.1 ± 2.5
**Left RF**	328.8 ± 77.5	319.8 ± 75.0	17.4 ± 2.7	17.1 ± 2.7	1.0 ± 0.2	0.9 ± 0.2	15.9 ± 3.3	15.9 ± 3.1
**Right RA**	265.4 ± 59.2	239.3 ± 54.4	14.7 ± 1.8	14.1 ± 1.9	1.2 ± 0.2	1.3 ± 0.4	18.9 ± 3.1	20.4 ± 2.9
**Left RA**	245.2 ± 60.6	254.4 ± 64.4	14.5 ± 2.0	14.7 ± 1.9	1.1 ± 0.3	1.2 ± 0.3	19.6 ± 2.9	19.2 ± 2.9
**Right VM**	252.5 ± 79.9	227.3 ± 68.9	15.2 ± 2.4	14.1 ± 2.0	0.8 ± 0.1	0.8 ± 0.2	19.1 ± 3.9	20.3 ± 3.4
**Left VM**	252.7 ± 60.1	235.2 ± 49.5	15.1 ± 1.6	14.6 ± 1.3	0.8 ± 0.1	0.7 ± 0.1	18.8 ± 3.0	19.2 ± 2.2

**Note:**

Dm, decrement; BB, biceps brachii; PM, pectoralis major; D, deltoid; I, infraspinatus; Abd, rectus abdominis; BF, biceps femoris; RF, rectus femoris; VM, vastus medialis; G, gastrocnemius. Data are mean ± SD.

**Table 4:** Postural control differences pre-post-match-play (*n* = 17).

Variable	PRE	POST	ES	Change (%)
COP Displacement EO (mm)	487.2 ± 227.1	583.1 ± 356.5	−0.47	19.7
COP Displacement EC (mm)	692.1 ± 406.4	752.6 ± 496.9	−0.13	8.7
COP Surface Area EO (mm)	201.3 ± 180.1	314 ± 289.1	−0.56	55.9
COP Surface Area EC (mm)	246.9 ± 183.5	326.2 ± 296.2	−0.44	32.1
Average Speed of COP EO (mm·s^−1^)	9.5 ± 4.4	11.3 ± 7	−0.48	18.9
Average Speed of COP EC (mm·s^−1^)	13.5 ± 7.9	14.7 ± 9.7	−0.14	8.9

**Note:**

COP, center of pressure; EO, eyes open; EC, eyes closed; ES, Cohen’s effect size. Data are mean ± SD.

## Discussion

The main finding of this study was that a single simulated tennis match did not significantly affect mechanical muscle characteristics or postural control in young tennis players. Certain trends and changes could be observed resulting in slightly decreased stiffness and tone and increased time to relaxation in the right rectus abdominis. Also, non-significant decreased tone seemed to appear in the right vastus medialis. Although moderate changes appear in total surface area during a balance test, postural control indicators of fatigue at the central level seem generally unaffected by 80 min of competition.

Previous research has shown the appearance of central and peripheral fatigue indicators following tasks of different duration and intensities (Macgregor et al., 2016; Sadeghi, Newman & Cortes, 2018; Thomas et al., 2015). Specifically in tennis, it seems clear that isometric strength and ROM around the hip and shoulder complex are negatively affected following match-play (Gallo-Salazar et al., 2017; Gescheit et al., 2015). Thus, although performance seems altered due to fatigue, it would seem these changes should be caused or accompanied by variations in muscle characteristics and therefore alterations in stiffness or other parameters (Macgregor et al., 2016; Sadeghi, Newman & Cortes, 2018). This is given to happen as high-intensity activities might trigger coupling impairment, destruction of cellular structures or membrane integrity due to exercise-induced muscle damage (Hunter et al., 2012). Nonetheless, no significant changes in mechanical properties of the muscles tested were found in this study. The right rectus abdominis and vastus medialis were the only muscles that showed certain decreases in stiffness and tone and an increase in time to relaxation in post-match measurements. The rectus abdominis is placed in great stress during tennis match-play. Its role is of extreme importance in the kinetic chain present in tennis strokes (Roetert et al., 2009) and activation can be elevated. Specially in the serve or during overhead strokes, eccentric activity is important to support the trunk and avoid excessive spinal stress. Subsequently, during the acceleration phase, a counter rotation occurs, eliciting high concentric activity of the trunk flexors and rotators (Correia et al., 2016). Furthermore, abdominal muscles play a key role as co-contraction muscles in pelvic rotation, leg raising and in cutting maneuvers (Whyte et al., 2018). Also, we should consider the high level of participation of the vastus medialis during the serve, specially at the end of the concentric phase (Girard, Micallef & Millet, 2005). During side-step pivoting and braking patterns it behaves as a stabilizer muscle that could increase fatigue levels (Brown, Brughelli & Hume, 2014). Because of these demands on the mentioned muscles, mechanical characteristics might have been affected and tone and stiffness would follow other usual fatigue responses and suffer alterations. Nevertheless, results can be surprising taking into account that redistribution of sarcomere lengths, the loss of membrane integrity or the destruction of cellular structures are contributors to enhanced tone and stiffness (Macgregor et al., 2016) and not reductions as happens here. One of the reasons could be that these muscles are eccentrically stimulated repetitively during tennis competition during long periods of time. The high stretch-shortening cycle (SSC) nature of tennis actions can place a certain load on the aforementioned muscles, and an impairment in SSC performance can be associated with a reduced muscle and joint stiffness, due to muscle damage induced by the eccentric phase of the SSC itself (Kuitunen et al., 2002). As seen previously, this might elicit inflammatory responses and edema, which may represent a protective mechanism designed to combat the load associated with long duration activities (Andonian et al., 2016), and decrease stiffness and tone levels in consequence. Nevertheless, the activity pattern and load placed on players here seems rather dissimilar to that presented by Andonian et al. (2016). In fact, as presented in Table 2, match-load characteristics, especially those concerning external load measurements, are in accordance to matches of similar duration and population (Gallo-Salazar et al., 2019; Hoppe et al., 2014). While accelerations, decelerations, total distance and velocity indicators are similar to comparable interventions, internal load results (i.e., peak and mean heart rate) seem of a smaller magnitude. Mean values of registered matches indicated 144.7 ± 12.5 beats·min^−1^ and time spent in zones 1 and 2 (<85% HR_max_) accounted for approximately 65% of playing time. Therefore, generally non-altered tone and stiffness values might be, in fact, due to low stress or load placed on the subjects. Following this idea, Thomas et al. (2015) found that peripheral fatigue indicators presented lower values following longer tasks than in shorter and intense bouts, possibly due to factors other than muscle fatigue playing a larger role in limiting and reducing performance. An 80-min simulated match at a mean HR of 144.7 beats·min^−1^ and 65% of playing time below 85% of HR_max_ might respond to a moderate intensity task and therefore derive in the observed results. Hence, duration and intensity could be determinants to show changes in the tested variables. Previous research using electromyographic activity as a fatigue indicator observed negative effects on MVC after 180 min of tennis match-play. Although no measurements where performed mid-match for this variable in the mentioned study, maximal joint force reductions seemed to appear at 90 min of play (Martin et al., 2016). In spite of the fact that effective playing time was different in the mentioned investigation and measurements accounted for MVC values, results may indicate that peripheral muscle fatigue indicators might appear with higher loads and that an 80-min simulated match would be insufficient for mechanical property markers to be sensitive to track fatigue, if present. Moreover, RPE registered in Martin et al. (2016) accounted for 12.5 ± 1.9 after 90 min of competition whereas 80 min of match-play resulted here in RPE scores of 15.2 ± 1.14, suggesting further data registered in longer or more strenuous competitions would be of great interest. In this line, some investigations observed how mechanical muscle properties are affected alongside other fatigue indicators. For instance, Wiewelhove et al. (2017) found TMG markers not sensitive for monitoring fatigue in youth tennis players following a high-intensity training (HIT) microcycle, although other peripheral fatigue indicators such as counter movement jump (CMJ) performance and muscle soreness (DOMS) were negatively affected. As the authors point out, the fact that results involving TMG markers did not follow other fatigue indicators could be explained by the fact that younger athletes seem to present a smaller extent of exercise-induced muscle damage than adults due to a strategy of the central nervous system to limit the recruitment of muscle units to prevent any extensive peripheral fatigue (Ratel et al., 2015). Taking this into account and considering the load of the training protocol in Wiewelhove et al. (2017) of a greater magnitude than 80 min of match-play presented here, muscle mechanical parameters might have not been sensitive to the load presented as this has to be of a greater magnitude or applied repetitively, especially when referring to adolescents.

Regarding central fatigue indicators, in our study we hypothesized that metabolic and perceptual fatigue caused by a simulated tennis match would negatively influence stability as tennis players must handle both physiological and cognitive loads during match-play (Davranche & Pichon, 2005). As found in similar investigations (Malliou et al., 2010), our results suggest a trend towards worsening, but no significant differences could be observed following the intervention. In general, results regarding the center of pressures (COP), average speed or total displacement of the COP indicate that a competition of these characteristics does not seem to affect the tested parameters. This data should be interpreted with caution because some factors should be taken into consideration. First, fatigue seems to influence the central nervous system differently depending on the duration, type of physical exercise and level of coordination (Tomporowski, 2003). In the same way as in our study, Douchamp-Riboux (1989) did not observe any modification in afferent systems functionality when analyzing a sub-maximum type of physical activity such as a rowing marathon. Meanwhile other studies seem to indicate that incremental exhausting exercises increase the sensory threshold, suggesting an increased cortical arousal as a compensatory pathway (Davranche & Pichon, 2005), as an ability of the central nervous system to tolerate a greater peripheral fatigue. Second, the absence of significant differences in the stability variables does not necessarily mean no effects on the stability pattern in the upright stance, because different strategies for balance control could be active. These strategies could be a different ratio between: i) the activation level of the proximal (hip) and distal (ankle) muscles that will involve different muscle patterns (Henry, Fung & Horak, 2001), ii) the loss or limitation of the information provided by one afferent source could be replaced with the increase of another sensory input from a different source (Tchórzewski, Bujas & Jankowicz-Szymańska, 2013) or iii) key muscular proprioceptive systems are not altered by an activity of these characteristics (Malliou et al., 2010), in accordance to muscle contractile properties found here. Nevertheless, moderate trends showed a reduction in stiffness and tone in the rectus abdominis muscle, which are evidently important due to its influence on balance (Larson & Brown, 2018). Although non-significant, the moderate-to-large increase in the displacement of COP with eyes open found in this study might follow the idea that balance was affected to some extent after a load of these characteristics, but one of the mentioned compensatory pathways might appear in order to maintain balance control. As seen previously, postural control can be recovered in relatively short periods of time following fatiguing exercises, due to the body’s capacity to adapt gathering other somatosensory information to function appropriately (Larson & Brown, 2018). In any case, data suggests that the match induced load could have been moderate and of an insufficient magnitude to show further variations.

This investigation had some limitations. First, the non-inclusion of inter-session reliability measurements and/or a control group could affect results. Second, previously, performance factors have been shown to decrease after competition (Gallo-Salazar et al., 2017; Gescheit et al., 2015), whereas muscle mechanical characteristics seem unaffected by higher intensity activities (Wiewelhove et al., 2017). The analysis of physical factors alongside other peripheral and central system fatigue indicators would have given insight on the effect of the proposed load in this study on further performance aspects. Also, comparisons with other investigations have to be interpreted with caution due to the fact that the competition here was in simulated conditions and physical and physiological outcomes significantly vary depending on the type of performance. In this line, match load characteristics were registered for 10 subjects only, while the sample size was higher. Last, fatigue at the central level was indirectly assessed using postural control measurements, while directly related methods could have offered different results.

## Conclusions

In conclusion, an 80-min simulated tennis match does not seem to trigger significant changes in muscle characteristics and postural control. Data suggests that physiological responses induced by these match conditions were closer to those following a moderate activity than carrying out a strenuous task.

## Supplemental Information

10.7717/peerj.11445/supp-1 Supplemental Information 1 Raw data: Analyzed variables.

## Additional Information and Declarations

### Data Availability

The following information was supplied regarding data availability: Raw measurements are available in the Supplemental File.
